# Peritoneal metastases from malignant degeneration of ectopic gastric epithelium in Meckel’s diverticulum: A case report

**DOI:** 10.1016/j.ijscr.2019.07.005

**Published:** 2019-07-12

**Authors:** Ehab El Bahesh, Bruce M. Abell, Paul H. Sugarbaker, Antoine Finianos, Marilyn Baird-Howell, James D. Ahlgren

**Affiliations:** a Division of Hematology and Oncology, George Washington University Cancer Center, Washington DC, USA; b Department of Surgery, George Washington University Medical Center, Washington DC, USA; c Department of Surgery, Washington Hospital Center, Washington DC, USA; d Department of Pathology, George Washington University Medical Center, Washington DC, USA

**Keywords:** Meckel diverticulum, Metastatic adenocarcinoma, O-MAX chemotherapy, Complete remission

## Abstract

**Background:**

Meckel’s diverticulum is the most common congenital anomaly of the gastrointestinal tract, resulting from incomplete obliteration of the omphalomesenteric duct in early fetal development. The diverticulum frequently contains ectopic gastrointestinal epithelial tissue, most commonly gastric or pancreatic. Adenocarcinoma can arise from either of these epithelia but is extremely rare, with less than 50 cases reported in the world literature.

**Methods:**

In a patient with peritoneal metastases from ectopic gastric mucosae in a Meckel’s diverticulum a multidisciplinary treatment plan was initiated.

**Results:**

Diagnosis was evident from study of the resected specimen of small bowel in a patient with acute abdominal pain. A multiagent chemotherapy regimen using oxaliplatin, mitomycin C, doxorubicin, and capecitabine (O-Max) resulted in a clinical and radiologic response. A second surgery with extensive sampling of tissues from the abdomen and pelvis confirmed a complete response to systemic chemotherapy. At four years from onset of disease the patient has no evidence of recurrence.

**Conclusions:**

Peritoneal metastases from malignant degeneration of ectopic gastric epithelium in a Meckel’s diverticulum was reported. Multidisciplinary treatments were beneficial.

## Highlights

• Meckel’s diverticulum is an outpouching of the distal small bowel.
• This diverticulum may contain ectopic gastric epithelium which can become malignant.
• A patient with gastric cancer with peritoneal metastases had the cancer origin in a Meckel’s diverticulum.
• Systemic cancer chemotherapy followed by cytoreductive surgery provided 4 years of disease-free survival.

## 1 Introduction

The Meckel’s diverticulum was named after Johann Friedrich Meckel who described the embryological origin of this type of diverticulum in 1809 [1]. It is a remnant of omphalomesenteric (vitelline) duct which connects the distal small bowel to the placenta during embryonic development. This important connection is within the ventral mesentery, the great majority of which does not persist during fetal development. The omphalomesenteric duct narrows and disappears between the fifth and eighth weeks of gestation. For a Meckel’s diverticulum to form the proximal part of the duct based on the terminal ileum fails to involute. The remnant lies on the antimesenteric border of the ileum and directly opposite the small bowel mesentery. The diverticulum is consistently located 60–100 cm from the ileocaecal valve and is 3–6 cm in length. The diameter of the lumen is variable and may be as great or greater than the diameter of the terminal ileum. Meckel’s diverticulum is the most common anomaly of the gastrointestinal tract and present in 2% of adults [2].

The surgical and oncologic relevance of Meckel’s diverticulum comes from its pluripotent cell lining. Jejunal mucosa, duodenal mucosa with Brunner’s glands, rests of gastric mucosa (60%) and pancreatic tissue (6%) may be present within the diverticulum [3]. This ectopic tissue may undergo malignant degeneration. Because the wall of the diverticulum is thin, a small cancer can disseminate itself widely within the peritoneal space. Cancers in a Meckel’s diverticulum report to date include pancreas cancer and gastric cancer [[4], [5], [6]].

In this case report a malignancy within the Meckel’s diverticulum caused peritoneal metastases of gastric cancer tissue type. To our knowledge this is the first patient with gastric cancer peritoneal metastases reported in the literature and successfully treated. A continuum of treatments has led to an unusual outcome with gastric cancer peritoneal metastases long-term survival.

Data on this patient was prospectively recorded and then retrospectively reviewed at an academic institution. This research work has been reported in line with the PROCESS criteria [7]. This study was registered as a case report on the www.researchregistry.com website with UIN 4835.

## 2 Case presentation

**Fig. 1:**
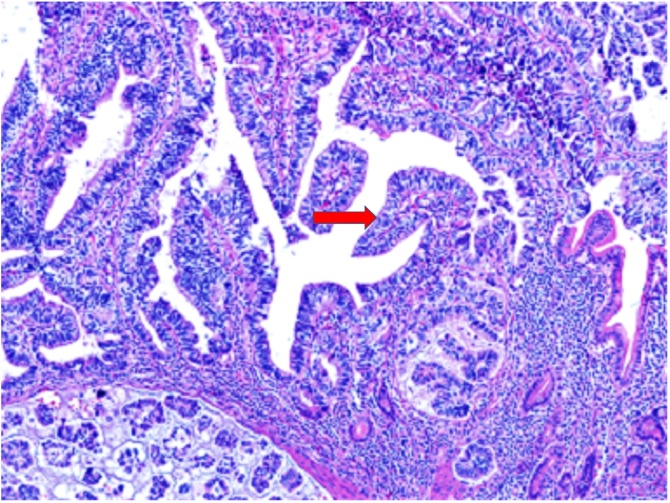
Adenocarcinoma of Meckel’s Diverticulum. Extensive gastric epithelial differentiation (arrow) strongly suggests origin in ectopic gastric epithelium.

A 45-year-old man presented to the Emergency Department with severe abdominal pain. CT revealed diffuse inflammation of the right lower quadrant, with a tubular structure arising from the small bowel and nodularity in the omentum. He was taken urgently to exploratory laparotomy revealing a mass arising in an inflamed Meckel's diverticulum and surrounded by extensive peritoneal metastases involving particularly the cecum, right colon, sigmoid and descending colon, and omentum. The diverticulum, containing a 3.5-cm tumor, was resected along with a 10-cm segment of ileum. Multiple areas of peritoneal studding were sampled and the omentum was biopsied. Pathology revealed invasive moderately differentiated adenocarcinoma involving all specimens. The proximal small bowel resection margin was involved. Tumor cells were positive for CK7, MOC31, BerEP4, CK19 and CA 19-9. Staining was negative for CK20, CK30, PAP, PSA, WT-1, CK5/6, inhibin, OCT3/4, and calretinin. This immunohistochemical profile, along with the presence of gastric epithelial differentiation, favored origin in ectopic gastric epithelium (Fig. 1).

He made a routine postsurgical recovery but his pain persisted. Because there were no apparent hematogenous metastases, he was considered to be a candidate for cytoreductive surgery (CRS) with heated intraperitoneal chemotherapy (HIPEC), which has been shown to be effective in some patients with carcinomatosis arising from a variety of primary malignancies [8], including gastric cancer [9].

Prior to undertaking cytoreductive surgery (CRS) with hyperthermic intraperitoneal chemotherapy (HIPEC), neoadjuvant systemic chemotherapy (NAC) was to be administered with the goal of reducing the extent of the metastatic disease. We chose the O-MAX regimen, which has shown very high activity in metastatic gastric carcinoma, particularly with respect to objective response rate and rate of complete response (10). CT prior to chemotherapy showed nodularity in the omentum and extensive peritoneal studding, most prominent in the right lower quadrant. He received two courses (16 weeks) of O-MAX, which was delivered at a dose intensity of 94%. His pain resolved. At the end of the chemotherapy, CT showed no evidence of omental or peritoneal tumor.

Four weeks after completion of systemic chemotherapy, the patient underwent CRS. He underwent open exploration, with careful examination of the entire abdominal cavity. There was no visible evidence of cancer but layers of fibrosis in areas of prior cancer. The surgical strategy was to resect all anatomic sites layered by scar tissue to resect chemotherapy-resistant cells. He underwent total omentectomy, resection of a segment of ileum on either side of the previous small bowel resection with re-anastomosis. A wide lymph node dissection of the small bowel mesentery was performed. There was a resection of the scar from the previous abdominal incision, and extensive biopsies of peritoneal adhesions including an 8-cm wide strip of anterior parietal peritoneum in the vicinity of the old abdominal incision. He then received HIPEC with cisplatin, doxorubicin intraperitoneally and ifosfamide by continuous intravenous infusion during the 90 min of hyperthermia [8]. Final pathologic examination of all specimens from the second surgery (prior to HIPEC) showed no evidence of viable tumor. He made a rapid postoperative recovery. Four years later he remains without evidence of recurrent disease.

## 3 Discussion

### 3.1 Management strategies for gastric cancer with peritoneal metastases

The results of treatment of gastric cancer with peritoneal metastases are poor. Perhaps the most favorable report comes from the French Association of Surgery multi-institutional study [11]. These patients were treated with cytoreductive surgery and a perioperative hyperthermic intraperitoneal chemotherapy. The median survival was 9.2 months and five-year survival of 13%. Gill et al. published a systematic review of CRS and HIPEC with gastric cancer patients with peritoneal metastases. Median survival was 7.9 months and 13% of patients were alive at five years [12].

Currently, three different treatment strategies are being pursued in gastric cancer patients with peritoneal metastases. Perhaps the most common is the strategy being studied by Rau et al. in a randomized controlled trial [13]. After NAC, cytoreductive surgery is performed. Then patients are randomized to receive HIPEC with cisplatin and mitomycin C. Results from this trial are not yet reported. Yang et al. has reported a randomized study with HIPEC in patients having cytoreduction. Median survival in the surgery only group was 6.5 months and it the surgery plus HIPEC group 11.9 months [14].

A second strategy is neoadjuvant intraperitoneal chemotherapy using paclitaxel [15] or docetaxel plus cisplatin [16]. The chemotherapy is administered through an intraperitoneal port using repeated instillations. The extent of peritoneal metastases is monitored by serial laparoscopy performed approximately every 3 months. If there is a robust response and cytology becomes negative, gastrectomy and cytoreduction of gastric cancer peritoneal metastases can result in 20% long-term survivors.

The third strategy is often referred to as the American approach [17]. The multidisciplinary team evaluates the patient to choose a systemic chemotherapy that is thought to be appropriate for a particular patient. The most aggressive gastric cancer treatment regimen that the patient can tolerate is selected. In our patient in this case report the O-MAX regimen was selected [10]. If the response to the systemic chemotherapy is complete or near complete, a gastrectomy to resect the primary disease site and cytoreduction to remove residual sites of peritoneal metastases may be recommended. The recommendation for surgery is a composite of the patient’s fitness for surgery and the response to gastric cancer chemotherapy. The beneficial effects from the systemic chemotherapy are usually obvious as performance status improves. CT assessment of the volume of disease is crucial. If there are doubts regarding the adequacy of the chemotherapy response, laparoscopy is indicated.

In our patient the American approach was used. After the diagnosis was established, neoadjuvant chemotherapy with an aggressive systemic chemotherapy regimen was begun. As a result of subjective, clinical and radiologic evidence of a robust response, a surgical exploration and then cytoreductive surgery was performed. Sites of possible disease persistence were removed and extensive sampling of tissue occurred.

### 3.2 Neoadjuvant systemic chemotherapy with O-MAX

**Fig. 2:**
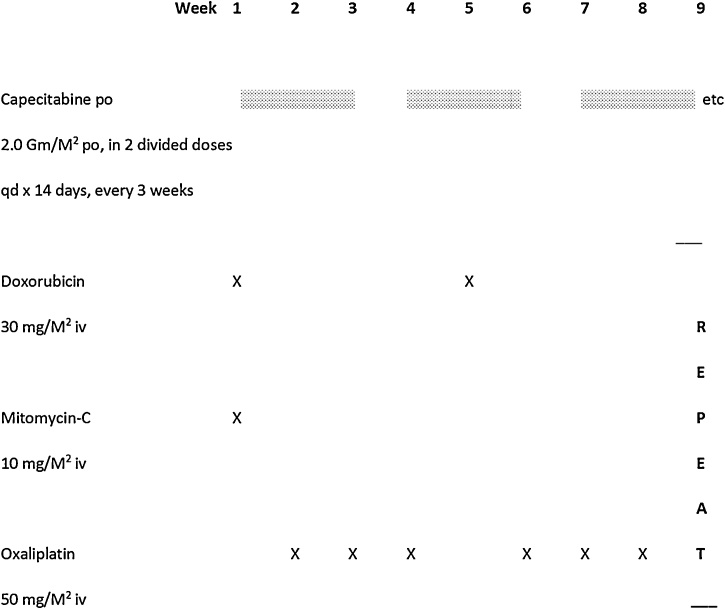
O-MAX Regimen.

O-MAX is an intensive 8-week regimen for gastric and esophageal adenocarcinoma, combining mitomycin-C, doxorubicin, oxaliplatin, and capecitabine (Fig. 2). In the first 25 consecutive patients (all metastatic) treated with O-MAX for adenocarcinoma of the stomach or esophagus, the objective response rate was 90% with 38% complete remissions [10]. Preliminary evidence suggested subclinical hemolysis in complete responders, implying the presence of antitumor antibodies as identified by Camtrell et al. [18]. Median survival was 16.5 months and three long-term survivors remain in complete remission without maintenance. This patient, with pre-planned cytoreductive surgery, presented a unique opportunity for pathologic confirmation of the complete response of his metastatic cancer to O-MAX. The case demonstrates that, when origin in ectopic gastric epithelium can be demonstrated, adenocarcinoma originating in Meckel's diverticulum can be a chemosensitive tumor. It also reinforces the exceptionally high activity of O-MAX in gastric carcinoma.

From Ref. [10].

## Sources of funding

Secretarial support and data management support funded by Foundation for Applied Research in Gastrointestinal Oncology.

## Ethical approval

Local IRB-approval for this case report was not required:

MedStar Health Institutional Review Board has determined that a case report of less than three (3) patients **does not meet the DHHS definition of research** (45 CFR 46.102(d)(pre-2018)/45 CFR 46.102(l)(1/19/2017)) **or the FDA definition of clinical investigation** (21 CFR 46.102(c)) and therefore are not subject to IRB review requirements and **do not require IRB approval**.

This case report is of 1 patient.

## Consent

Written informed consent was obtained from the patient for publication of this case report and accompanying images. A copy of the written consent is available for review by the Editor-in-Chief of this journal on request.

## Author’s contribution

Paul H. Sugarbaker, MD: study concept or design, data collection, data analysis or interpretation, writing the paper.

Ehab El Bahesh, MD: study concept or design, data collection, data analysis or interpretation, writing the paper.

Bruce M. Abell, MD: study concept or design, data collection, data analysis or interpretation, writing the paper.

Antoine Finianos, MD: study concept or design, data collection, data analysis or interpretation, writing the paper.

Marilyn Baird-Howell, MD: study concept or design, data collection, data analysis or interpretation, writing the paper.

James D. Ahlgren, MD: study concept or design, data collection, data analysis or interpretation, writing the paper.

## Registration of research studies

This case report is registered as a case series on the www.researchregistry.com website with UIN 4835.

## Guarantor

Paul H. Sugarbaker, MD.

## Provenance and peer review

Not commissioned, internally peer-reviewed.

## Declaration of Competing Interest

The author has no conflicts of interest to declare.
